# MRI markers of glymphatic dysfunction in tuberculous meningitis: associations with CSF proteins and cognitive impairment

**DOI:** 10.3389/fneur.2026.1772389

**Published:** 2026-06-05

**Authors:** Yilin Wang, Wei He, Yongbo Li, Dailun Hou

**Affiliations:** 1 Department of Radiology, Beijing Tuberculosis and Thoracic Tumor Research Institute, Beijing, China; 2 Department of Radiology, Beijing Chest Hospital, Capital Medical University, Beijing, China; 3 Department of Radiology, Shaanxi Provincial Tuberculosis Prevention and Control Hospital The Fifth People’s Hospital of Shaanxi Province, Xi’an, Shaanxi, China

**Keywords:** choroid plexus, cognitive function, diffusion tensor imaging along the perivascular space, glymphatic system, tuberculous meningitis

## Abstract

**Objectives:**

Tuberculous meningitis (TBM), a central nervous system infection, is frequently complicated by mild cognitive impairment (MCI). Although imaging markers of glymphatic function have been shown to correlate with cognitive decline, its role in TBM remains unrevealed. This study aims to examine the associations among glymphatic function, cognitive performance, and cerebrospinal fluid proteins in patients with TBM using functional magnetic resonance imaging (fMRI).

**Methods:**

This study enrolled 123 participants (61 healthy controls (HCs), 30 TBM-nonMCI, 32 TBM-MCI) who underwent MRI scanning and cognitive assessments. The perivascular space (PVS) load, diffusion-tensor imaging along the perivascular spaces (DTI-ALPS) index and choroid plexus volume (CPV) were calculated to evaluate lymphatic function. CSF proteomics were analyzed in TBM patients. Then, partial correlation and mediation analyses examined the associations among CSF proteins, lymphatic metrics, and cognition. Receiver operating characteristic curves were used to evaluated the predictive capacity for TBM-MCI diagnosis and 6-to 12-month treatment outcomes.

**Results:**

Compared with HCs, all patients showed smaller hippocampal PVS volume fraction and larger CPV, and the TBM-MCI group exhibited lower ALPS and higher basal ganglia PVS. ALPS indices positively correlated with cognitive measures (SDMT, MMSE), while hippocampal PVS positively correlated with multiple cognitive domains; CPV exhibited opposite correlations. The right CPV fully mediated the relationship between the right ALPS-MMSE and the right ALPS index, distinguishing TBM-MCI from HCs (AUC = 0.719). The left ALPS index predicted a 6- to 12-month cognitive prognosis (AUC = 0.857), with CSF proteins not mediating glymphatic-cognitive relationships.

**Conclusion:**

The association between imaging markers suggestive of altered perivascular clearance and cognitive decline in tuberculous meningitis underscores the potential mechanistic roles of the ALPS and CPV, positioning the ALPS index as a promising biomarker candidate for prognosis and cognitive dysfunction.

## 1 Introduction

Tuberculous meningitis (TBM) represents one of the most severe central nervous system (CNS) infections, frequently leaving survivors with substantial cognitive impairment (CI) and diminished long-term quality of life (1). Although extensive research has been conducted, the pathophysiological mechanisms underlying TBM-associated cognitive deficits remain incompletely understood. Conventional explanations focusing on direct parenchymal injury, vasculitis, and intracranial hypertension (2) fail to fully account for the heterogeneity in CI severity and patterns, particularly during the early mild CI (MCI) stage—a critical window for therapeutic intervention. This gap underscores the need to identify novel, broadly applicable neurobiological pathways to inform effective treatments.

Emerging evidence highlights the glymphatic system (GS)—a brain-wide perivascular network responsible for clearing metabolic waste—as a pivotal mechanism in cognitive decline across various neurological disorders (3). Notably, in multiple neurological disorders—such as Alzheimer’s disease (AD) (4), and idiopathic normal-pressure hydrocephalus (5)—disrupted CSF flow and meningeal inflammation have been associated with impaired glymphatic transport and contribute to cognitive decline. These findings suggest that CSF circulation abnormalities may represent a convergent pathway linking neuroinflammation to cognition across disease entities. We thus hypothesize that chronic inflammation, meningeal thickening, and cerebrospinal fluid (CSF) flow disturbances in TBM disrupt glymphatic circulation, impair clearance of neurotoxic proteinss, and thereby drive cognitive decline, particularly during the MCI phase.

Non-invasive magnetic resonance imaging (MRI) biomarkers now enable *in vivo* assessment of glymphatic function (GF). Among these, the diffusion tensor image analysis along the perivascular space (DTI-ALPS) index quantifies glymphatic transport efficiency and has been validated in AD (6, 7), cerebral small vessel disease (CSVD) (8, 9), Parkinson’s disease (PD) (10), and traumatic brain injury (TBI) (11), where lower values correlated with more severe cognitive deficits. In addition, enlarged perivascular spaces (PVS) reflect glymphatic outflow obstruction, with basal ganglia PVS (PVS-BG) associated with aging and hypertension (3), white matter PVS (PVS-WM) linked to amyloid deposition and neurodegeneration (3), and hippocampal PVS(PVS-Hipp) changes related to memory impairment and AD (6), PD pathology (10). Furthermore, choroid plexus volume (CPV), indicative of upstream glymphatic regulation, shows enlargement in settings of neuroinflammation and blood-CSF barrier (BBB) disruption, correlating with poorer cognitive outcomes in multiple sclerosis and AD (12–14). However, these methods have been rarely applied in TBM.

While imaging provides macroscopic insights into GS, underlying molecular mechanisms in CSF remain largely unexplored. As a direct interface with brain interstitial fluid, CSF serves as a unique biological window. Growing evidence connects glymphatic impairment (GI) to reduced Aβ/tau clearance (15), inflammatory cytokines (e.g., IL-6, IL-16) that compromise BBB integrity (16), and vascular/lymphatic regulators (e.g., LYVE-1, ANGPTLs) that modulate perivascular dynamics (17–19). Guided by this foundation, we incorporated high-throughput CSF proteomics to identify molecular drivers of GS in TBM.

To guide interpretation of the imaging markers used in this study, we propose a conceptual framework distinguishing three interrelated but distinct aspects of pathology in TBM:

1. Inflammatory injury: Direct neuroinflammation caused by *Mycobacterium tuberculosis* infection leads to blood–brain barrier disruption, glial activation, and tissue damage. This is captured indirectly by CPV, which enlarges in response to neuroinflammation and blood-CSF barrier disruption.
2. Vascular and perivascular alterations: Meningeal inflammation and vascular pathology in TBM affect arterial pulsatility and perivascular flow dynamics. These changes are reflected in PVS burden, particularly in the basal ganglia and white matter, where enlarged PVS indicate impaired perivascular drainage.
3. Clearance-related proxy measures: The net efficiency of interstitial fluid and waste clearance along perivascular pathways is proxied by the DTI-ALPS index, which quantifies water diffusivity along the perivascular direction. A lower ALPS index suggests reduced perivascular transport capacity.

Together, these markers provide complementary views of the glymphatic system and its associated pathology. CPV reflects upstream inflammatory status, PVS burden reflects downstream drainage obstruction, and the ALPS index captures the functional consequence of these alterations on perivascular clearance. This framework informs the hypothesized pathway (CP → ALPS → cognition) tested in our mediation analysis.

In this longitudinal study, we integrated the DTI-ALPS index, PVS, and CPV across to: (1) compare GF among groups; (2) examine correlations and mediation effects among glymphatic metrics, cognitive scores, and CSF proteins levels; (3) evaluate their utility in distinguishing TBM patients at risk of MCI; and (4) assess GF as a predictor of cognitive prognosis in TBM. We hypothesize that GD is associated with increased risk of TBM-related MCI and may serve as a predictive indicator for cognitive outcomes.

## 2 Methods

### 2.1 Study population

The study cohort consisted of 123 participants, including 62 patients with TBM and 61 healthy controls (HCs). Patients with TBM were recruited from the inpatient department of Beijing Chest Hospital, Capital Medical University. Age-, sex-, and education-level–matched HCs were recruited from the community. The inclusion criteria for both patients with TBM and HCs were as follows: (1) age 18–60 years; (2) education at primary school level or above; (3) right-handedness; (4) absence of MRI contraindications; and (5) no history of psychiatric or neurological disorders. According to established diagnostic criteria (20, 21), TBM was diagnosed in patients who met any of the following conditions: with a clinical diagnosis of TBM, i.e., at least 5 days of meningitis symptoms, and cerebrospinal fluid (CSF) abnormalities; with anti-tuberculosis chemotherapy already started or planned by the treating clinician.

**Figure 1:**
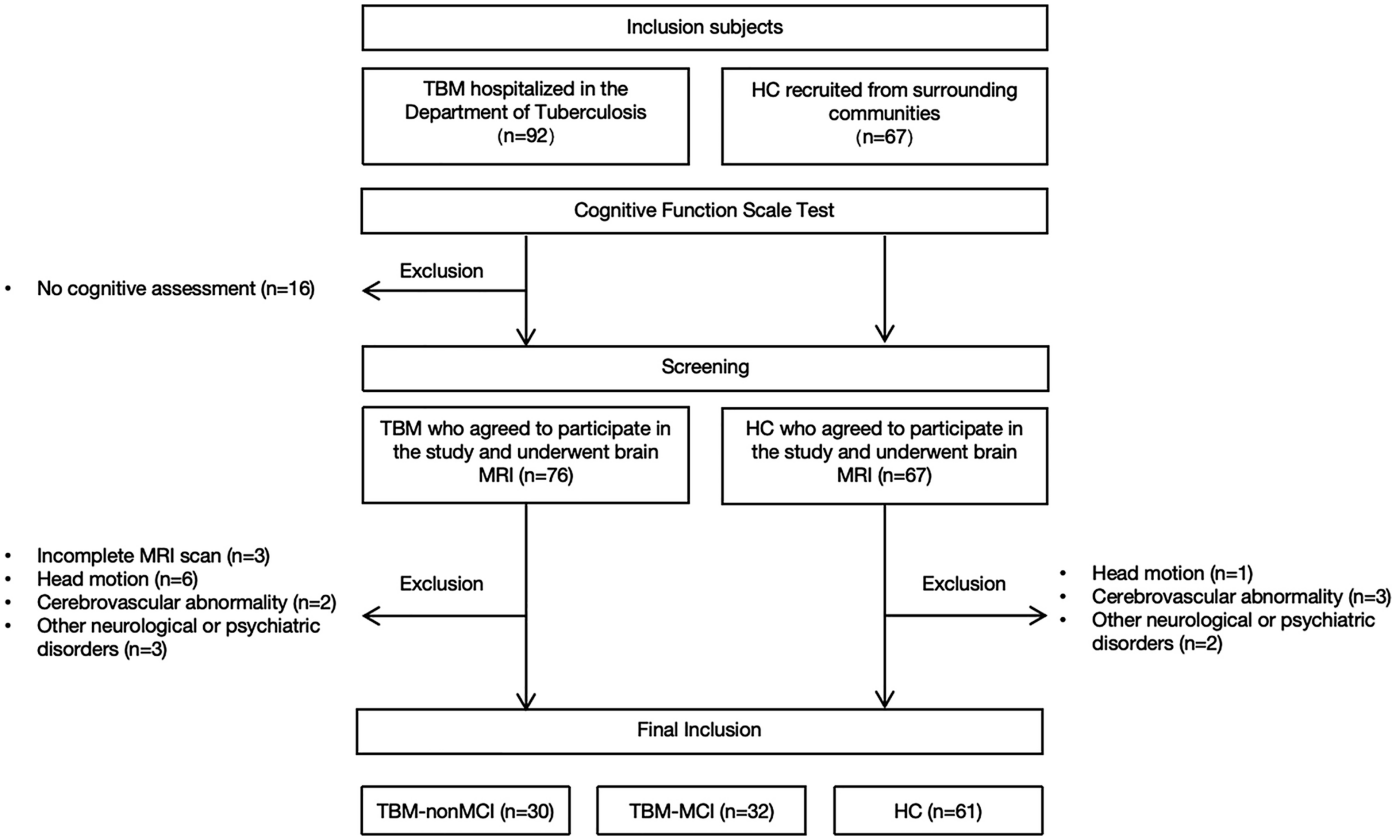
Flowchart of participants included in the study. TBM, Tuberculous Meningitis; HC, healthy controls; MRI, magnetic resonance imaging; MCI, mild cognitive impairment; TBM-nonMCI, TBM patients with normal cognition; TBM-MCI, TBM patients with MCI.

The general exclusion criteria for patients with TBM and HCs were as follows: (1) a history of substance (drug, nicotine, or alcohol) abuse; (2) pregnancy or lactation; (3) concomitant neurological, cardiovascular, cerebrovascular, or endocrine disorders; (4) infection with human immunodeficiency virus; (5) hearing, visual, or physical impairments that precluded completion of cognitive or imaging assessments; and (6) poor MRI data quality (e.g., significant susceptibility artifacts or incomplete raw data). Specifically, patients with a history of traumatic brain injury, stroke, or other neurological disorders that might affect glymphatic function were also excluded from the study. This study was conducted in accordance with the Declaration of Helsinki (2013 revision) and was approved by the Ethics Committee of Beijing Chest Hospital (BJXK-2024-KY-16). All participants provided informed consent. The flow diagram of data acquisition is shown in Figure 1.

Standardized diagnostic and initial treatment protocol: all patients were enrolled at the time of initial diagnosis, prior to the initiation of anti-tuberculous therapy. The diagnosis of TBM was established based on consistent clinical, CSF, and neuroimaging findings, and was confirmed by CSF microbiological tests (e.g., Xpert MTB/RIF, culture). Upon study enrollment and completion of all assessments, all patients uniformly received a standardized first-line anti-tuberculous regimen consisting of isoniazid, rifampin, pyrazinamide, ethambutol, and a fluoroquinolone, combined with adjunctive dexamethasone according to national guidelines. For patients presenting with elevated intracranial pressure, osmotic agents (e.g., mannitol) were administered as clinically indicated.

All patients were enrolled at the time of initial diagnosis, prior to the initiation of anti-tuberculous therapy or corticosteroids. Baseline MRI scanning and cognitive assessments were performed on the same day, with cognitive testing conducted in a quiet room within the radiology department immediately before the MRI scan. This ensures that our baseline imaging and cognitive data reflect the native disease state without confounding by treatment effects.

### 2.2 Cognitive assessment

All participants completed a series of cognitive tests in a quiet room of the radiology department. Trained researchers administered all cognitive assessments before the study to minimize potential bias. The Mini-Mental State Examination (MMSE) and Montreal Cognitive Assessment (MoCA) were employed to assess general cognitive function comprehensively (22, 23). Executive function, attention, and cognitive flexibility were evaluated using Parts A and B of the Trail Making Test (TMT-A and TMT-B) (24). Visuospatial ability was assessed using the Clock Drawing Test (CDT) (25). The Verbal Fluency Test (VFT) was administered to evaluate verbal ability and executive control (26). The Digit Span Test (DST) was used to assess attention and working memory capacity (27). The Rey Auditory Verbal Learning Test (RAVLT) was administered to assess verbal memory for both immediate and delayed recall (26). Finally, the Symbol Digit Modalities Test (SDMT) was administered to assess information processing speed (26).

Participants were classified into cognitive groups based on a comprehensive assessment that operationalized the core principles of the Petersen criteria for MCI (28) and the DSM-5 criteria for Mild Neurocognitive Disorder (29). These frameworks require: (1) evidence of cognitive decline from a previous level, (2) objective impairment in one or more cognitive domains, (3) preservation of functional independence, and (4) absence of dementia. Grouping criteria for MCI were based on scores from the MMSE and the MoCA, both of which have a maximum score of 30. For participants with fewer than 12 years of formal education, one point was added to the MoCA score, as per standard guidelines. Participants were classified as having MCI if they scored <26 on the MoCA and between 21 and 26 on the MMSE. Individuals with MMSE scores greater than 27 and MoCA scores of 26 or higher were considered cognitively normal. In cases where MMSE and MoCA scores were inconsistent, MMSE results were prioritized, and the clinical features of MCI were additionally considered. These criteria included: (1) reports from family members or other informants indicating memory impairment as the main complaint, while other cognitive domains were either preserved or only mildly affected; (2) preserved activities of daily living that did not meet the diagnostic criteria for dementia; and (3) exclusion of other neurological or systemic disorders that could explain the observed cognitive decline.

To address the potential concern regarding the lower bound of the MMSE cutoff (21) and its possible overlap with early dementia, we conducted a sensitivity analysis using a more conservative definition. In this sensitivity analysis, the lower bound of the MMSE score was raised from 21 to 23, a threshold more commonly used to differentiate MCI from mild dementia (30). Participants were re-classified using this more stringent criterion while maintaining all other clinical requirements (informant report, preserved ADLs, exclusion of other causes).

Based on the cognitive assessment results, patients with TBM were divided into two subgroups: TBM-MCI and TBM-nonMCI.

### 2.3 MRI data acquisition

MRI scanning was performed using a 3 T Signa Architect (GE Healthcare) with a 48-channel phased-array head coil after neurocognitive tests. Diffusion tensor imaging (DTI), T1-weighted imaging (T1WI), and T2-weighted fluid-attenuated inversion recovery (T2-FLAIR) imaging data were acquired for each participant.

High-resolution three-dimensional T1-weighted magnetization-prepared rapid gradient-echo (3D-T1 MPRAGE) images were acquired at the beginning of each scanning session for anatomical registration [field of view (FOV) = 240 × 240 mm^2^; voxel size = 0.9 × 0.9 × 0.9 mm^3^; slice thickness = 0.6 mm; number of slices = 484; interslice gap = 0; repetition time (TR) = 7.9 ms; flip angle = 8°; acquisition time = 7 min 18 s]. DTI was performed using identical parameters on both scanners: FOV = 240 × 240 mm^2^; TR = 8,000 ms; echo time = minimum; matrix = 128 × 130; diffusion encoding directions = 30; slice thickness = 5 mm; number of slices = 29; interslice gap = 0; b-values = 0 and 1,000 s/mm^2^; acquisition time = 4 min 16 s. T2-weighted fluid-attenuated inversion recovery (T2-FLAIR) images were obtained for all patients to exclude other brain pathologies such as stroke or tumors [TR = auto; echo time (TE) = minimum; FOV = 240 × 240 mm^2^; slice thickness = 5 mm; number of slices = 29; interslice gap = 0]. Gadolinium-enhanced T1-weighted (Gd-T1) images were acquired for 62 patients to characterize brain lesions better, classify the type of tuberculous Meningitis, and assess ventricular enlargement.

### 2.4 Analysis of the DTI-ALPS index

**Figure 2:**
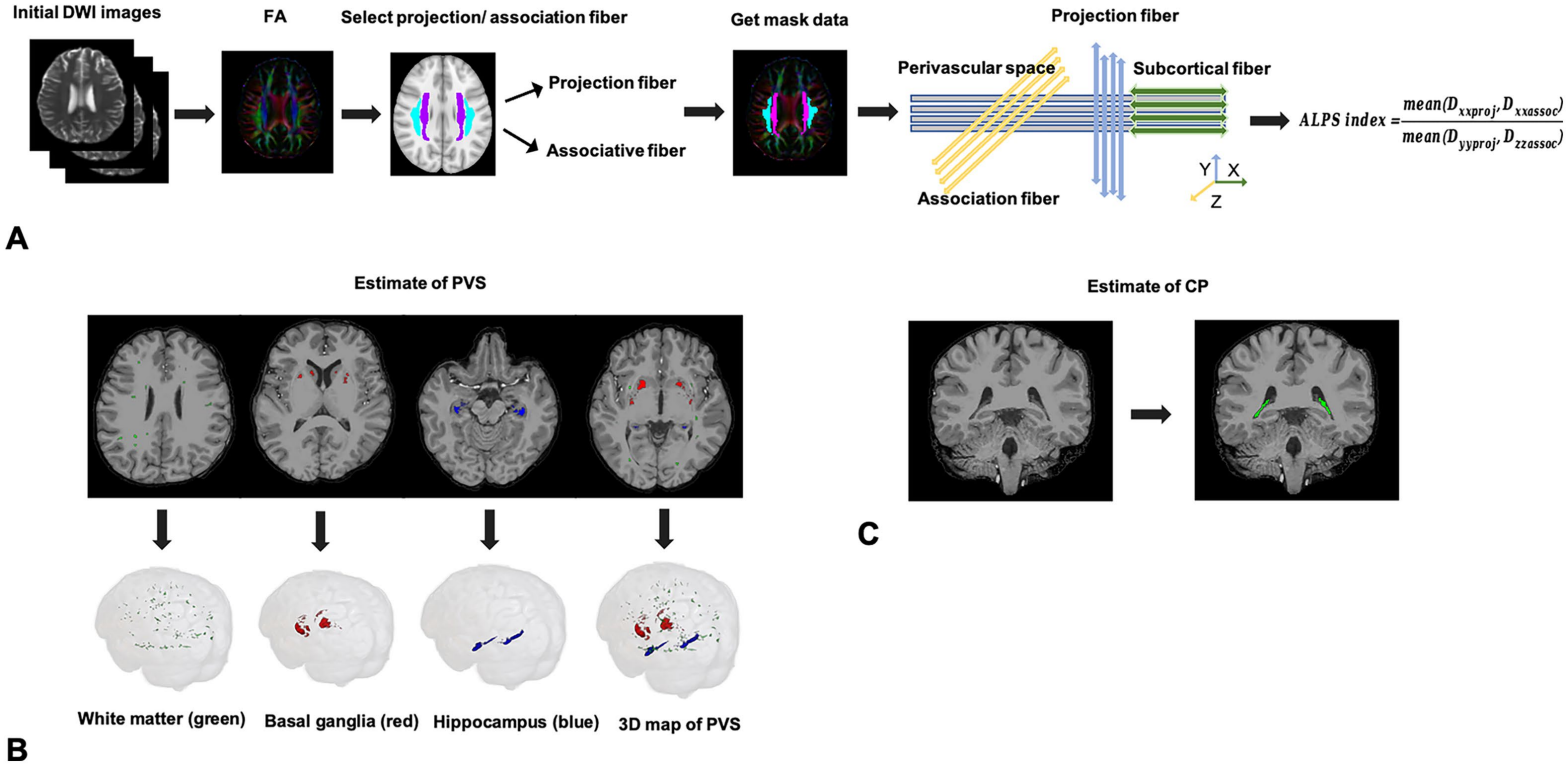
**(A)** ALPS index calculated; **(B)** PVS mapping, PVSs were then extracted from the white matter (green), basal ganglia (red), and hippocampus (blue); **(C)** CP segmentation. DWI, Diffusion weighted imaging; ALPS, analysis along the perivascular spaces; PVS, perivascular spaces; CP, choroid plexus.

In this study, the DTI-ALPS index was used as an indirect MRI marker of glymphatic transport efficiency, based on the premise that higher diffusivity along the perivascular direction reflects better preserved interstitial fluid clearance. DTI data were processed to quantify glymphatic activity using the analysis ALPS. Method, as shown in Figure 2A. The DTI-ALPS method enables the quantification of glymphatic activity along perivascular spaces by analyzing multidirectional diffusivity maps derived from DTI data. The DTI images were processed and modeled using the FMRIB Software Library,^1^ following these steps:

#### 2.4.1 Anterior commissure–posterior commissure alignment and reorientation

To correct for potential head tilt in raw images, the b0 image was rigidly aligned to a standard template using FSL’s FLIRT tool (six degrees of freedom: rotation and translation) (31), ensuring AC–PC alignment. The resulting linear deformation field was then applied to adjust the diffusion gradient directions accordingly.

#### 2.4.2 Skull stripping and brain extraction

Non-brain tissues (e.g., scalp, skull) were removed using FSL’s Brain Extraction Tool (BET) (32), generating a brain mask from the b0 image for subsequent processing.

#### 2.4.3 Preprocessing and denoising

Diffusion-weighted images (DWI) were preprocessed using MRtrix3 (33) and FSL. The processing steps included: PCA-based denoising to reduce random noise, Gibbs ringing correction to suppress truncation artifacts; eddy current and motion correction using FSL’s *eddy* tool (34); and ANTs-based N4 bias field correction (35) to address B₀ inhomogeneity.

#### 2.4.4 Diffusion tensor reconstruction

The diffusion tensor model was fitted using FSL’s dtifit (32), producing maps of fractional anisotropy (FA) and principal directional diffusivities (Dxx, Dyy, and Dzz).

#### 2.4.5 ALPS calculation

FA maps were registered to the ICBM DTI-81 standard space atlas via T1WI using FSL, with registration accuracy verified by visual inspection. The ICBM DTI-81 atlas contains labels for projection fibers (e.g., superior and posterior corona radiata) and association fibers (e.g., superior longitudinal fasciculus) in the periventricular region. Projection and association fibers within 25–33 mm above the AC–PC line in MNI space were extracted, as this region corresponds to the x-axis direction of penetrating vessels in the deep white matter. The ALPS index and diffusivity values for projection and association fibers were computed using the method described by Taoka et al. (7).

The ALPS index is calculated based on the average values of Dxx, Dyy, and Dzz extracted within the two ROIs through the following formula:

$$\text{ALPS index}=\frac{\text{mean}(D_{\text{xxproj},}D_{\text{xxassoc}})}{\text{mean}(D_{\text{yyproj}},D_{\text{zzassoc}})}$$

To ensure reproducibility, all ROI placements were independently verified by two trained raters (with 10 and 5 years of neuroimaging experience), who were blinded to clinical and cognitive data. Intra-rater reliability was assessed by having the same rater re-draw ROIs in a random subset of 20 participants after a 2-week interval. Inter-rater and intra-rater reliability for the ALPS index were excellent, with intraclass correlation coefficients (ICCs) of 0.92 and 0.95, respectively.

### 2.5 PVS quantification

PVS burden, quantified as PVS volume fraction (PVSVF) and PVS count in the basal ganglia, white matter, and hippocampus, was used as a surrogate marker of impaired perivascular drainage, based on the established interpretation that enlarged PVS reflect obstruction of glymphatic outflow pathways. Data analysis was performed using FreeSurfer (version 7.4.1). The software automatically delineates cortical structures, and all raw T1-weighted images underwent standard preprocessing before Formal analysis. The preprocessing steps included motion correction, removal of non-brain tissue, transformation to the Talairach template for spatial normalization, intensity normalization, 3D cortical surface reconstruction, cortical inflation, and spherical mapping. These procedures were used to obtain masks of white matter, subcortical nuclei, and other relevant regions.

The Frangi vesselness filter was applied to the preprocessed T1-weighted images to estimate vessel-like structures based on the eigenvectors of the Hessian matrix for each voxel. This operation was implemented using the QIT toolkit,^2^ with default parameters (*α* = 0.5, *β* = 0.5), while the parameter *c* was set to half of the maximum Hessian norm. The filter was applied across multiple spatial scales (0.1–5 voxels) to identify structures with the highest likelihood of perivascular features, generating PVS probability maps. PVSs were extracted from T1W images using a fully automated and validated quantification pipeline consistent with prior work (36, 37). The PVS volume within the white matter (WM), hippocampal (Hipp), and subcortical nuclear regions (basal ganglia, BG) was subsequently calculated, as shown in Figure 2B. Total intracranial volume (TIV) was calculated from whole-brain T1-weighted image segmentation using the Computational Anatomy Toolbox (CAT12), an extension of the Statistical Parametric Mapping software package.^3^ The PVSVF (PVSVF = PVS volume/ TIV) was then calculated to eliminate interindividual variability in brain size.

Quality Control and Reliability: All automated PVS segmentations underwent rigorous visual inspection by two trained raters blinded to group status. Segmentation quality was assessed using a 3-point scale (1 = poor, 2 = acceptable, 3 = good); only scans with a quality score ≥2 were included in the final analysis. In cases of obvious segmentation errors (e.g., misclassification of lacunes or enlarged perivascular spaces), manual corrections were performed using the editing tools in the QIT toolkit. Inter-rater reliability for PVSVF measurements, assessed in a random subset of 20 participants, yielded ICCs of 0.89 for white matter, 0.91 for basal ganglia, and 0.88 for hippocampus.

### 2.6 CP volume measurement

CPV with enlargement interpreted as suggestive of neuroinflammation and blood-CSF barrier disruption, is consistent with previous studies. The CP, the primary site of CSF secretion, is increasingly recognized as an indirect imaging biomarker of CSF production and toxic clearance (38, 39). Structural T1-weighted images were processed using FreeSurfer.^4^ Prior to formal brain imaging analysis, preprocessing of raw data for each subject was required, following the same steps as in the PVS section. As shown in Figure 2C, the CP within the lateral ventricles was automatically segmented from the T1-weighted images, which have been previously utilized for segmenting CP from structural MRIs (40, 41). The segmentation results underwent comprehensive visual inspection, with manual corrections applied where necessary. The final normalized CP volume was calculated by summing the bilateral CP volumes and dividing by the TIV.

Accounting for Potential Confounding by Ventricular Size: To ensure that CPV measurements were not confounded by ventricular enlargement, we carefully reviewed all baseline images for evidence of hydrocephalus. Importantly, no patients in this cohort had radiological evidence of hydrocephalus on baseline imaging, as confirmed by clinical radiology reports and visual inspection of T1-weighted and T2-FLAIR images by two experienced neuroradiologists. Therefore, ventricular enlargement was not a confounding factor for CPV measurements in this study. All CP segmentations were additionally verified to be located within non-dilated lateral ventricles, further confirming the absence of hydrocephalus-related effects on CPV estimates.

### 2.7 Quantitative proteomic analysis

CSF samples were collected from patients with TBM within 2 days after MRI acquisition at Beijing Chest Hospital. Under sterile conditions, approximately 1 mL of CSF supernatant was obtained during lumbar puncture, filtered through a 0.22-μm membrane, aliquoted into 0.5 mL per tube, and stored at −80 °C until analysis. The complete procedures for quantitative proteomic analysis are described in the Supplementary material.

### 2.8 Statistical analysis

All statistical analyses were performed using SPSS version 26.0 (IBM Corp., Armonk, NY, United States). The Shapiro–Wilk test was first applied to assess the normality of continuous variables. Normally distributed variables were expressed as mean ± standard deviation (mean ± SD). Intergroup differences in demographic, clinical, and glymphatic-related imaging parameters (ALPS index, PVS, and CPV) among the TBM-MCI, TBM-nonMCI, and HC groups were compared using one-way analysis of variance (ANOVA) followed by Bonferroni *post hoc* tests. Non-normally distributed variables were presented as medians (interquartile ranges, IQRs) and compared using the Kruskal–Wallis test with the Bonferroni correction. Categorical variables were summarized as counts (percentages), and differences among groups were assessed using the chi-square test or Fisher’s exact test, as appropriate. A false discovery rate (FDR) of 0.05 was applied using the Benjamini–Hochberg approach for correction of multiple comparisons when appropriate. Demographic variables, including age, sex, and years of education, were included as covariates in all group comparisons and regression models involving cognitive or imaging outcomes to control for potential confounding. For analyses specifically focusing on volumetric brain structure outcomes, such as PVSVF and CPV, TIV was included as a covariate to correct for individual differences in head size.

For all other analyses, including those involving the DTI-ALPS index (a diffusivity ratio) and cognitive scores, TIV was not included as a covariate, as these measures are not inherently dependent on head size.

Covariates and Confounding Control: In addition to demographic variables (age, sex, years of education) and TIV, we further considered potential disease-specific confounders.

- Disease Severity: TBM severity was graded according to the British Medical Research Council (MRC) staging system (stage I, II, or III) based on level of consciousness and neurological deficits at presentation. As shown in Table 1, MRC stage did not differ significantly between the TBM-MCI and TBM-nonMCI groups (*p* > 0.05), suggesting that disease severity was comparable across patient subgroups. Nevertheless, to ensure robustness, MRC stage was included as an ordinal covariate in sensitivity analyses for the primary correlation models.
- Ventricular Enlargement and Hydrocephalus: Hydrocephalus is a known complication of TBM that could potentially affect perivascular spaces and glymphatic measures. However, in the present cohort, no patients presented with radiological evidence of hydrocephalus on baseline imaging (as determined by the absence of disproportionate ventricular enlargement relative to sulcal size). Therefore, ventricular size was not included as a covariate, as it showed no variability across the sample.
- Focal Lesion Burden: Tuberculomas and infarcts are common in TBM and may influence both imaging metrics and cognition. However, given the heterogeneity in lesion location, size, and number across patients, a simple quantitative score may not adequately capture their complex effects. As an alternative, we conducted sensitivity analyses excluding patients with large (>1 cm) tuberculomas or territorial infarcts that could disproportionately affect regional measurements. The results remained consistent with the main findings (data not shown), suggesting that our primary observations were not driven by focal lesions.

**Table 1:** Demographics and clinical characteristics of patients with TBM and HCs.

Demographics	HC (*n* = 61)	TBM-nonMCI (*n* = 30)	TBM-MCI (*n* = 32)	*p* value			
				HC vs. TBM-nonMCI vs. TBM-MCI	HC vs. TBM-nonMCI	HC vs. TBM-MCI	TBM-nonMCI vs. TBM-MCI
Age (year)	43.11 (14.82)	39.53 (13.89)	44.97 (15.16)	0.335			
Gender (male/female)	32/29	18/12	20/12	1.000			
Education (year)	12.39 (2.04)	12.23 (2.14)	11.41 (1.97)	0.084			
TIV (cm^3^)	1438.20 (130.98)	1443.240 (149.182)	1409.911 (119.365)	0.609			
GCS grade		30	32	–	–	–	0.237
I (> = 13)	–	16	23				
II (9–12)	–	10	4				
III (<=8)	–	4	5				
Treatment							
Standardized anti-tuberculous	–	30	32				0.237
Dexamethasone		17	15				0.079
Osmotic agents		5	6				0.432
MMSE	30.00 (29.00–30.00)	28.33 (0.96)	25.00 (22.25, 25.75)	0.000	0.222	0.000	0.000
MoCA	26.92 (2.78)	26.03 (2.33)	21.16 (4.62)	0.000	0.448	0.000	0.000
TMT-A (s)	33.00 (25.00, 44.00)	38.00 (28.75, 44.50)	63.69 (21.26)	0.000	0.571	0.000	0.000
TMT-B (s)	81.00 (64.00, 115.00)	79.00 (66.00, 108.25)	159.72 (72.02)	0.000	0.603	0.002	0.001
CDT	4.89 (0.32)	4.70 (0.75)	4.19 (0.97)	0.000	0.415	0.000	0.007
VFT	44.80 (11.63)	37.43 (10.22)	33.09 (6.53)	0.000	0.004	0.000	0.219
DST forward	10.89 (2.56)	11.87 (1.28)	9.59 (1.97)	0.000	0.108	0.019	0.000
DST backward	8 (5.50, 10)	9.10 (1.99)	5.84 (1.46)	0.000	0.174	0.000	0.000
SDMT	50.44 (13.29)	46.43 (8.34)	32.75 (8.18)	0.000	0.239	0.000	0.000
CSF proteins							
MMP19	–	2864.27 (2597.96)	2885.75 (2782.57)	–	–	–	0.622
MMP9		2864.27 (2597.96)	2885.75 (2782.57)	–	–	–	0.975
ICAM1	–	309.34 (96.94)	325.25 (137.34)	–	–	–	0.221
VCAM1	–	1313.58 (498.76)	1468.08 (612.60)	–	–	–	0.369
APLP1	–	3969.45 (1242.94)	4597.71 (2661.50)	–	–	–	0.094
ANGPTL2	–	85.55 (23.28)	81.27 (33.13)	–	–	–	0.136
TNFRSF1B	–	19.56 (18.67)	22.42 (16.67)	–	–	–	0.940
TGFB1	–	80.89 (93.76)	69.89 (80.10)				0.848

Normal distribution data are presented as mean (SD); non-normal distribution data are presented as median (interquartile range); HC, healthy controls; MCI, mild cognitive impairment; standardized anti-tuberculous regimen consisting of isoniazid, rifampin, pyrazinamide, ethambutol, and a fluoroquinolone, combined with adjunctive dexamethasone according to national guidelines; osmotic agents (e.g., mannitol); MMSE, mini-mental state examination; MoCA, montreal cognitive assessment; TMT, trail making test; CDT, clock drawing test; VFT, verbal fluency test; DST, digital span test; SDMT, symbol-digit modalities test; TIV, total intracranial volume; *p < 0.05.

It is important to note that the categorization of TBM patients into MCI and nonMCI subgroups was performed only to characterize the study population and for initial group-wise comparisons of imaging metrics. For all subsequent analyses investigating the relationships between glymphatic function, molecular markers, and cognition—including partial correlation, mediation, and regression analyses—cognitive test scores were treated as continuous variables.

Through partial correlation analysis, we assessed the correlations between cerebrospinal fluid lymphatic system imaging metrics, cognitive function, and cerebrospinal fluid protein levels.

Mediation Analysis: To explore the potential pathways linking glymphatic imaging metrics to cognitive function, we conducted mediation analysis using the PROCESS macro for SPSS (version 4.2, Model 4; 62). Given the cross-sectional nature of our data, these analyses are framed as statistical mediation rather than tests of causal pathways. All models were tested with 5,000 bootstrap samples to generate bias-corrected 95% confidence intervals (CIs) for indirect effects. An indirect effect was considered statistically significant if the 95% CI did not include zero.

For each mediation model, we specified:

X (independent variable): glymphatic imaging metric (e.g., ALPS index, CPV, PVS metrics).

M (mediator): another glymphatic imaging metric hypothesized to lie on the pathway.

Y (dependent variable): cognitive test score (e.g., MMSE, TMT-A).

Covariates: All models included age, sex, and years of education. For models involving volumetric brain structure measures (e.g., CPV, PVSVF) as X, M, or Y, TIV was additionally included as a covariate to control for individual differences in head size.

Coefficients: Unstandardized regression coefficients (B) are reported for all paths, along with their 95% CIs. The proportion of the total effect mediated (mediation effect percentage) was calculated as (indirect effect/total effect) × 100% for models with a significant indirect effect.

To examine the robustness of our findings and address the inherent directionality ambiguity of cross-sectional mediation, we also tested alternative models with the hypothesized mediator and independent variable swapped (e.g., ALPS index as mediator vs. ALPS index as independent variable). These results are presented alongside the primary models to inform the interpretation of directional plausibility. Finally, multiple linear regression analysis was used to identify independent predictors of cognitive function, adjusting for age, gender, and educational attainment. Receiver operating characteristic (ROC) curve analysis evaluated the predictive performance of lymphatic function indicators in distinguishing patients with TBM complicated by MCI and in forecasting TBM prognosis. For the prognostic analysis, cognitive improvement at the 6–12 month follow-up was defined using a composite endpoint based on both the MMSE and MoCA. For each patient, change scores (*Δ*) were calculated by subtracting the baseline score from the follow-up score for both scales. Patients were classified into two groups: “Improved” group: patients who showed an increase in both ΔMMSE and ΔMoCA scores from baseline to follow-up. “Not improved” group: patients who showed no change or a decrease in either or both of the scales. This composite definition was chosen to provide a more conservative and reliable indicator of genuine cognitive recovery than relying on a single screening tool.

Sensitivity Analysis for Cognitive Improvement: To test the robustness of our findings to the specific definition of improvement, we performed a sensitivity analysis using a more stringent threshold: improvement was defined as an increase of ≥2 points on both the MMSE and MoCAfrom baseline to follow-up. This threshold was chosen based on previous studies suggesting that a 2-point change represents a clinically meaningful improvement on these scales.

The selection of the Left ALPS index as the predictor for the ROC analysis was guided by *a priori* hypotheses regarding the role of left hemisphere glymphatic function in cognitive outcomes, and was confirmed by its significant univariate association with the prognostic grouping (*p* < 0.05). Other glymphatic metrics (Right ALPS, CPV, and PVS) did not show significant associations in this preliminary screening and were therefore not included in the ROC analysis to avoid overfitting and multiple testing issues. Bilateral *p*-values < 0.05 were considered statistically significant.

Code Availability: The custom scripts and processing pipelines used for image analysis in this study, including DTI preprocessing, ALPS index calculation, PVS quantification using the Frangi filter, CP volume extraction, and mediation analysis syntax, are available as online Supplementary material. Detailed instructions and parameter settings are provided within the code files to facilitate replication by other research groups.

## 3 Results

### 3.1 Demographic and clinical characteristics of study populations

This study included 61 HCs (HCs, 32 males, mean age, 43.11 ± 14.82 years), 62 TBM groups (30 TBM-nonMCI, 18 males, mean age, 39.53 ± 13.89 years; and 32 TBM-MCI, 20 males; mean age, 44.97 ± 15.16 years). No significant differences were observed among the three groups in terms of age, sex, years of education, TIV, or disease severity, as shown in Table 1.

On the VFT, HCs scored significantly higher than both TBM-MCI and TBM-nonMCI patients, whereas no significant difference was found between the TBM-MCI and TBM-nonMCI groups. Across all other neuropsychological assessments, the TBM-MCI group performed significantly worse than both the HCs and TBM-nonMCI groups. In contrast, no significant differences were detected between HCs and TBM-nonMCI groups. Although CSF biomarker data were unavailable for all participants, complete datasets were obtained for 70% of TBM-MCI and 83% of TBM-nonMCI groups.

### 3.2 Sensitivity analysis for MCI classification

To evaluate the robustness of our MCI classification and address the potential concern regarding the lower bound of the MMSE cutoff (21), we performed a sensitivity analysis using a more conservative definition. In this analysis, the MMSE threshold for MCI classification was raised from ≥21 to ≥23, a cutoff more commonly employed to distinguish MCI from mild dementia.

Remarkably, when applying this more stringent criterion, all 32 patients originally classified as TBM-MCI remained within the MCI category, and all 30 patients in the TBM-nonMCI group remained classified as cognitively normal. No participant crossed groups as a result of the revised cutoff. This indicates that, in our cohort, no patient had an MMSE score in the 21–22 range that would have been reclassified as non-MCI under the more conservative definition.

Consequently, all subsequent group comparisons, including demographic characteristics, neuropsychological performance, MRI indicators, and correlation analyses, remained identical to those obtained using the original classification. The consistency of findings across different MMSE cutoffs demonstrates the stability of our grouping and suggests that any potential concern regarding misclassification at the lower MMSE boundary does not apply to this cohort. The complete results of the primary analyses are presented in sections 3.3–3.6 below, which reflect the unchanged group composition.

### 3.3 Intergroup differences in MRI indicators outcomes

**Figure 3:**
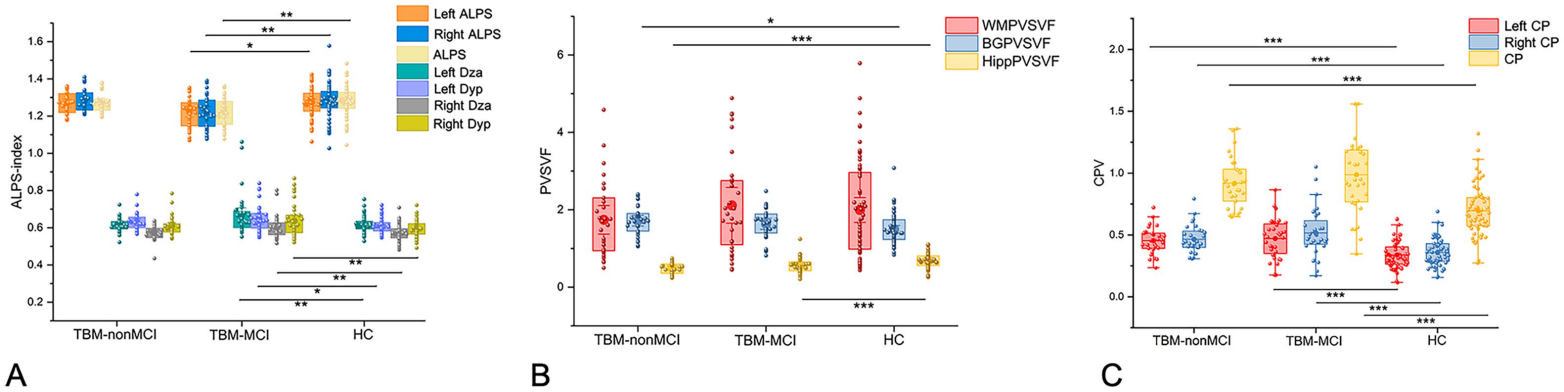
Intergroup differences in lymphatic indicators among the TBM-nonMCI, TBM-MCI, and IHC groups. **(A)** Group differences in the ALPS index and bilateral Dza and Dyp. **(B)** Group differences in the PVSVF index. **(C)** Group differences in CP volume. TBM, tuberculous meningitis; MCI, mild cognitive impairment; TBM-nonMCI, TBM patients with normal cognition; TBM-MCI, TBM patients with MCI; HC, healthy controls; ALPS, analysis along the perivascular spaces; Dza, diffusivity along the *z*-axis in association fiber area; Dyp, diffusivity along the *y*-axis in projection fiber area; PVSVF, perivascular spaces volume fraction; WM, white matter; BG, basal ganglia; Hipp, hippocampus; CP, choroid plexus, *p < 0.05, **p < 0.01, ***p < 0.001. For all figures, statistical significance was assessed using appropriate tests as described in the Methods section. For group comparisons, *p < 0.05, **p < 0.01, ***p < 0.001, indicates significance after FDR correction (*q* < 0.05). For correlation analyses, correlation coefficients (r) are reported. Units: ALPS index is unitless; PVSVF is expressed as percentage (%); CPV is normalized to TIV and expressed as mm^3^/cm^3^. Error bars represent standard deviation (SD) unless otherwise specified.

The results of intergroup comparisons are shown in Table 2 and Figure 3. All reported *p*-values have been corrected for multiple comparisons using the FDR method (Benjamini-Hochberg procedure, *q* < 0.05) to control for Type I error across the multiple imaging metrics examined. The left, right, and mean ALPS indices in the TBM-MCI group were significantly lower than those in the HC group (*p* = 0.029, 0.007, and 0.006, respectively). In contrast, the left and right Dzzassoc (*p* = 0.007 and 0.006) and Dyyproj indices (*p* = 0.015 and 0.003) were significantly higher in the TBM-MCI group compared with HCs. The left and right Dzzassoc indices (*p* = 0.025 and 0.017) were also considerably higher in the TBM-MCI than in the TBM-nonMCI group.

**Table 2:** Biomarkers of the glymphatic system in the participants.

	HCs	TBM	TBM-MCI	*p* value			
	*n* = 61	*n* = 30	*n* = 32	HC vs. TBM vs. MCI	HC vs. TBM	HC vs. MCI	TBM vs. MCI
Left Dxxproj	0.79 (0.04)	0.80 (0.06)	0.80 (0.08)	0.552			
Left Dyyproj	0.61 (0.58, 0.63)	0.62 (0.60, 0.66)	0.65 (0.08)	0.007	0.065	0.015	1
Left Dxxassoc	0.77 (0.05)	0.78 (0.04)	0.78 (0.75, 0.82)	0.163			
Left Dzzassoc	0.61 (0.04)	0.61 (0.04)	0.63 (0.60, 0.69)	0.006	1	0.007	0.025
Right Dyyproj	0.59 (0.04)	0.61 (0.05)	0.61 (0.57, 0.67)	0.003	0.331	0.003	0.367
Right Dxxproj	0.76 (0.04)	0.78 (0.06)	0.78 (0.08)	0.246			
Right Dxxassoc	0.73 (0.05)	0.73 (0.04)	0.71 (0.69, 0.76)	0.821			
Right Dzzassoc	0.57 (0.04)	0.58 (0.55–0.60)	0.58 (0.56, 0.63)	0.005	1	0.006	0.017
Left ALPS index	1.27 (0.07)	1.27 (0.06)	1.22 (0.08)	0.034	1	0.029	0.288
Right ALPS index	1.28 (0.09)	1.26 (1.23, 1.33)	1.22 (0.08)	0.008	1	0.007	0.056
ALPS index	1.28 (0.08)	1.27 (0.05)	1.22 (0.08)	0.008	1	0.006	0.079
PVS-WM number	190.00 (113.75, 216.75)	147.00 (102.00, 218.00)	172.72 (71.12)	0.044	0.045	0.591	1
PVSVF-WM(10^−3^%)	2.00 (1.25)	1.78 (0.99)	2.11 (1.29)	0.534			
PVS-BG number	15.15 (3.91)	13.68 (3.32)	14.75 (4.57)	0.138			
PVSVF-BG(10^−3^%)	1.49 (0.39)	1.71 (0.37)	1.64 (0.40)	0.018	0.014	1.000	0.322
PVS-Hipp number	5.00 (4.00, 6.75)	5.65 (2.24)	5.31 (2.19)	0.003	0.005	0.067	1.000
PVSVF-Hipp (10^−3^%)	0.68 (0.19)	0.49 (0.15)	0.52 (0.42, 0.68)	0.000	0.000	0.018	0.099
Left CP	0.338 (0.109)	0.454 (0.107)	0.471 (0.160)	0.000	0.000	0.000	1.000
Right CP	0.358 (0.109)	0.465 (0.113)	0.516 (0.195)	0.000	0.000	0.000	0.838
CP	0.697 (0.203)	0.919 (0.200)	0.987 (0.335)	0.000	0.000	0.000	1

Normal distribution data are presented as mean (SD); non-normal distribution data are presented as median (interquartile range); HCs, healthy controls; MCI, mild cognitive impairment; ALPS, analysis along the perivascular space; PVS, perivascular spaces; WM, white matter; BG, basal ganglia; Hipp, hippocampus; CP, choroid plexus. Dxxassoc/Dzzassoc, diffusivity along the x-axis/z-axis in association fiber area; Dyyproj/Dxxproj, diffusivity along the y-axis/x-axis in projection fiber area; diffusivity was presented as apparent diffusion coefficient values (× 10^−3^ mm^2^/s); *p < 0.05.

The overall TBM-MCI and TBM-nonMCI groups showed significantly lower PVS-WM counts (*p* = 0.045) but higher PVS-Hipp counts (*p* = 0.005) and PVSVF-BG values (*p* = 0.014) compared with HCs. Conversely, HCs exhibited higher PVSVF-Hipp values than both the TBM-nonMCI and TBM-MCI groups (*p* < 0.001 and *p* = 0.018, respectively). In both the TBM-nonMCI and TBM-MCI groups, the left CPV, right CPV, and CPV values were significantly higher than those in the HC group (all *p* < 0.001).

No statistically significant differences were found among the three groups in Dxxassoc, Dxxproj, PVSVF-WM, or PVS-BG counts. When comparing the TBM group with HCs, no significant differences were observed in Dzzassoc, Dyyproj, or the left, right, and whole-brain mean ALPS indices. When comparing TBM-MCI with HCs, no significant differences were observed in PVS-WM counts, PVS-Hipp counts, or PVSVF-BG values. When comparing TBM-MCI and TBM-nonMCI groups, no significant differences were found in Dyyproj, the left, right, and whole-brain mean ALPS indices, PVS-WM or PVS-Hipp counts, PVSVF-BG, PVSVF-Hipp, or the left, right, and whole-brain CPV measures.

Sensitivity Analyses: To assess the robustness of our findings, we performed sensitivity analyses adjusting for MRC stage and excluding patients with large focal lesions (>1 cm tuberculomas or territorial infarcts, *n* = 8). The significant correlations between glymphatic imaging metrics and cognitive scores remained largely unchanged after these adjustments (Supplementary Table 1), indicating that our findings are not attributable to differences in disease severity or the presence of focal parenchymal damage.

### 3.4 Relationship between imaging parameters, CSF protein levels, and cognitive function

**Figure 4:**
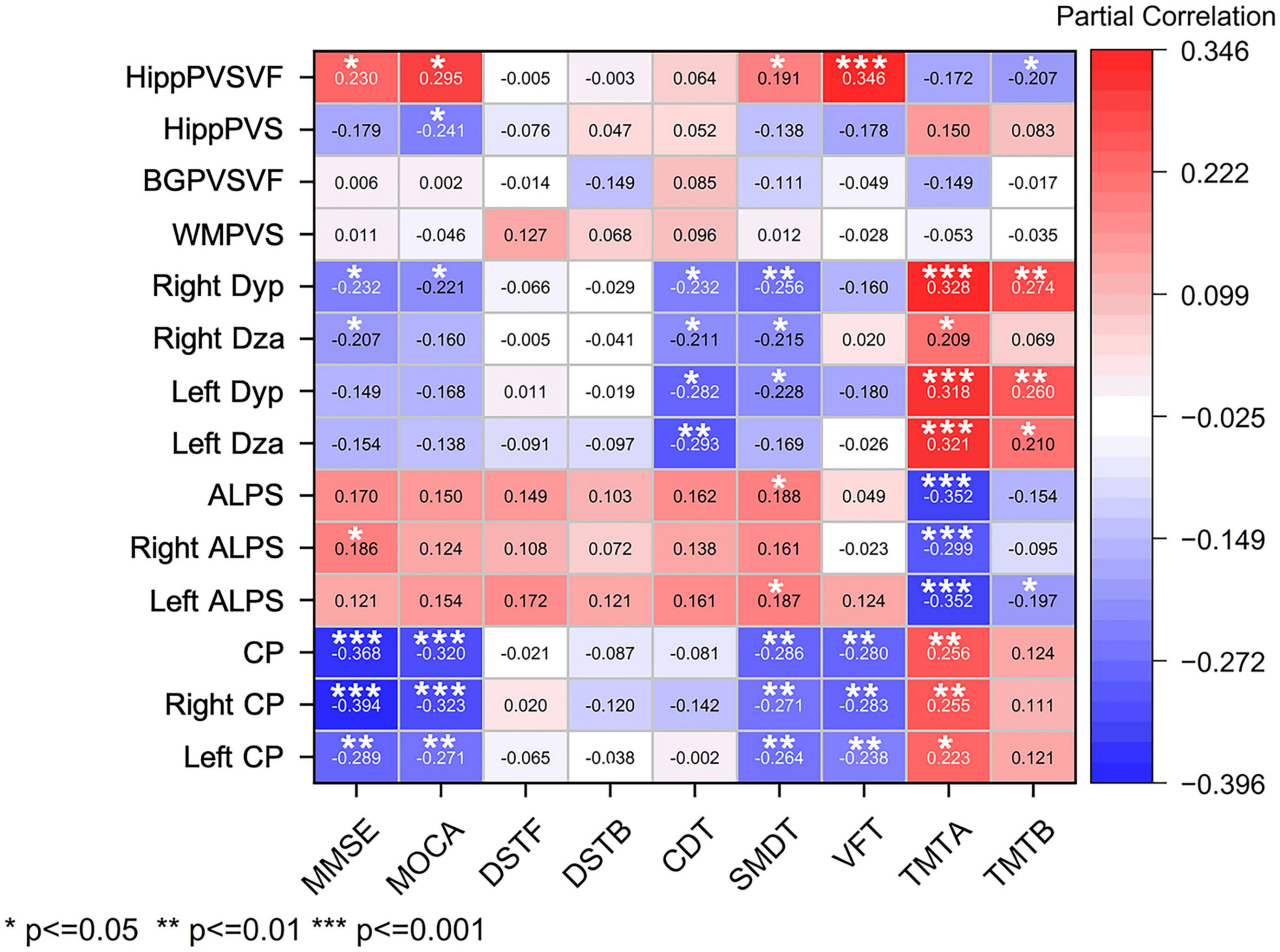
Correlation of lymphatic indicators with cognitive function. Red and blue indicate positive and negative correlations, respectively. ALPS, analysis along the perivascular spaces; Dyp, diffusivity along the *y*-axis in projection fiber area; Dza, diffusivity along the *z*-axis in association fiber area; PVS, perivascular spaces; PVSVF, PVS volume fraction; Hipp, hippocampus; BG, basal ganglia; WM, white matter; CP, choroid plexus; MMSE, Mini-Mental State Examination; MoCA, Montreal Cognitive Assessment; DSTF, Digit Span Test forward; DSTB, Digit Span Test backward; CDT, Clock Drawing Test; SDMT, Symbol Digit Modalities Test; VFT, Verbal Fluency Test; TMTA, Trail Making Test A part; TMTB, Trail Making Test B part. For all figures, statistical significance was assessed using appropriate tests as described in the Methods section. For group comparisons, *p < 0.05, **p < 0.01, ***p < 0.001, indicates significance after FDR correction (*q* < 0.05). For correlation analyses, correlation coefficients (r) are reported. Units: ALPS index is unitless; PVSVF is expressed as percentage (%); CPV is normalized to TIV and expressed as mm^3^/cm^3^. Error bars represent standard deviation (SD) unless otherwise specified.

Partial correlation analysis across all participants revealed the associations shown in Figure 4. p-values for these correlations have been corrected for multiple comparisons using the FDR method (*q* < 0.05). Correlations that remained significant after FDR correction are discussed below. Specifically, the study demonstrated the following associations: The left ALPS index was positively correlated with SDMT (*r* = 0.187) and negatively correlated with TMT-A (*r* = −0.352) and TMT-B (*r* = −0.197). The right ALPS index showed positive correlations with MMSE (*r* = 0.186) and SDMT (*r* = 0.188), whereas a negative correlation with TMT-A (*r* = −0.299), indicating that lower ALPS values were associated with poorer executive function.

The PVS-Hipp count was negatively correlated with overall cognitive performance, as measured by the MoCA (*r* = −0.241). The PVSVF-Hipp was positively correlated with global cognitive function (MMSE, *r* = 0.230; MoCA, *r* = 0.295), information processing speed (SDMT, *r* = 0.191), and verbal fluency (VFT, *r* = 0.346), while showing a negative correlation with executive function time (TMT-B, *r* = −0.207). The CPV in the left, right, and whole brain exhibited significant negative correlations with MMSE, MoCA, SDMT, and VFT scores (all *p* < 0.01), as well as a positive correlation with TMT-A (*p* < 0.05).

Among the lymphatic imaging biomarkers, we found that PVSVF-Hipp was negatively correlated with left CPV (*r* = −0.440), right CPV (*r* = −0.380), and CPV (*r* = −0.435; *p* < 0.001). The right ALPS Index was negatively correlated with the PVS-WM count (*r* = −0.261) and right CPV (*r* = −0.259; *p* < 0.05). Additionally, the PVS-WM count showed positive correlations with left CPV (*r* = 0.289), right CPV (*r* = 0.322), and CPV (*r* = 0.327) (*p* < 0.05). The PVS-Hipp count was positively correlated with left CPV (*r* = 0.386), right CPV (*r* = 0.294), and CPV (*r* = 0.359) (*p* < 0.05).

Significant associations were observed between CSF protein biomarkers, glymphatic imaging indices, and cognitive performance. VCAM1 showed a negative correlation with PVS-BG count (*r* = −0.493) and a positive correlation with MMSE scores (*r* = 0.654). APLP1 was positively correlated with PVSVF-BG (*r* = 0.558) and negatively correlated with VFT performance (*r* = −0.421). MMP9 demonstrated broad associations, showing negative correlations with BG-PVS count (*r* = −0.437) and positive correlations with PVSVF-Hipp (*r* = 0.482), MoCA scores (*r* = 0.428), and VFT scores (*r* = 0.553). MMP19 was negatively associated with relative CPV (*r* = −0.426) and PVS-BG count (*r* = −0.496), while positively associated with MMSE (*r* = 0.508). TNFRSF1B showed negative correlations with PVSVF-WM (*r* = −0.450) and positive associations with MMSE (*r* = 0.415). TGFB1 correlated positively with Left ALPS index (*r* = 0.432) and MMSE scores (*r* = 0.459), but negatively with PVS-BG count (*r* = −0.574); additionally, it was positively linked to DST-B performance (*r* = 0.471). ICAM1 showed negative correlations with left Dyypro (*r* = −0.454) and right Dyypro (*r* = −0.415), suggesting disrupted perivascular diffusivity.

### 3.5 Mediation analysis

To statistically disentangle the interrelationships among glymphatic imaging metrics and their joint association with cognitive function, we conducted mediation analysis using the PROCESS macro (Model 4) with 5,000 bootstrap iterations. Unstandardized regression coefficients (B) and bias-corrected 95% CI are reported. All models were adjusted for age, sex, and years of education; models involving volumetric measures were additionally adjusted for TIV.

**Figure 5:**
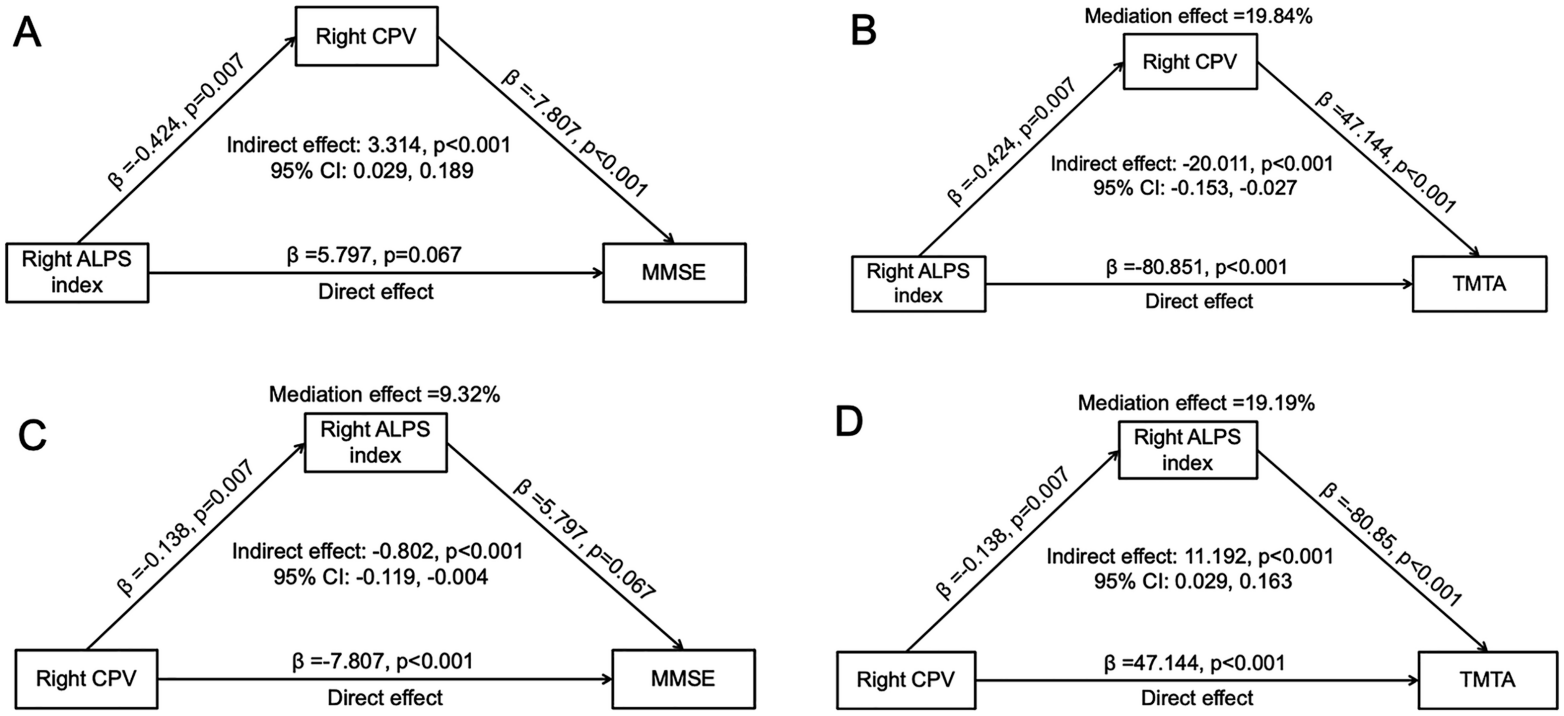
Mediation models. Path diagrams illustrate the mediating roles of right CPV and the right ALPS in the relationship between MMSE scores and TMT-A performance. **(A)** Full mediation by RCP. **(B–D)** Partial mediation. Right ALPS index, right cerebral hemisphere analysis along the perivascular spaces; Right CPV, right cerebral hemisphere choroid plexus volume; MMSE, mini-mental state examination; TMTA, trail making test A part. For all figures, statistical significance was assessed using appropriate tests as described in the Methods section. For group comparisons, *p < 0.05, **p < 0.01, ***p < 0.001, indicates significance after FDR correction (*q* < 0.05). For correlation analyses, correlation coefficients (*r*) are reported. Units: ALPS index is unitless; PVSVF is expressed as percentage (%); CPV is normalized to TIV and expressed as mm^3^/cm^3^. Error bars represent standard deviation (SD) unless otherwise specified.

The results of the mediation analysis are presented in Figure 5. To elucidate further the mediating effects of lymphoid-like imaging parameters on cognitive function, we conducted a mediation analysis. Right CPV fully mediated the relationship between the right ALPS index and MMSE score (indirect effect = 3.314, 95% CI: 0.029–0.189, *p* < 0.001). Right CPV also partially mediated the relationship between right ALPS index and TMT-A performance (indirect effect = −20.011, 95% CI: −0.153 to −0.027, *p* < 0.001; mediation effect = 19.84%).

Right ALPS index, in turn, partially mediated the relationship between right CPV and MMSE (indirect effect = −0.802, 95% CI: −0.119 to −0.004, *p* < 0.001; mediation effect = 9.32%) and between right CPV and TMT-A (indirect effect = 11.192, 95% CI: 0.029 to 0.163, *p* < 0.001; mediation effect = 19.19%).

No significant mediating effects of left, right, or whole-brain CPV were observed on the relationship between PVS-Hipp count and MoCA score. Similarly, no significant mediating effects were identified for left, right, or whole-brain CP on the relationships between PVSVF-Hipp and MMSE, MoCA, SDMT, or VFT scores.

### 3.6 Diagnostic and predictive value of lymphatic function for MCI

**Figure 6:**
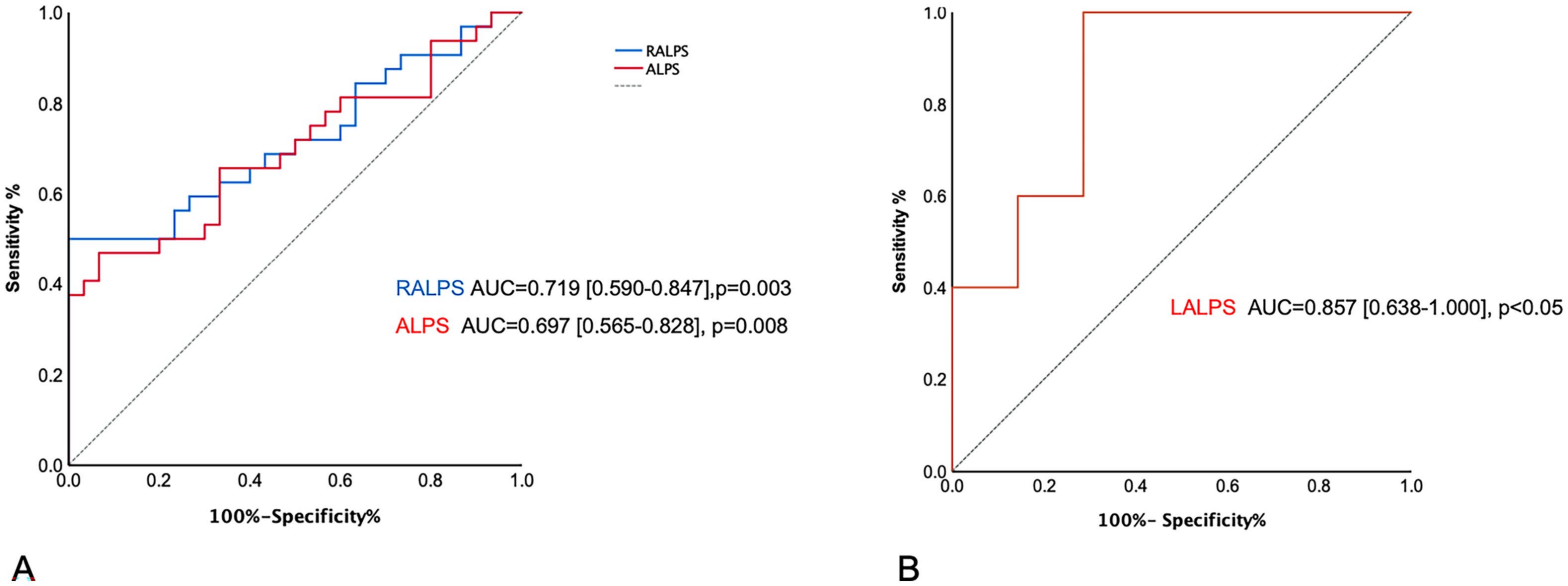
**(A)** ROC curves of the right ALPS index and ALPS index for predicting TBM with MCI. This assesses the predictive performance of each imaging indicator. **(B)** The left ALPS index in predicting 6–12 month cognitive improvement in TBM patients. Cognitive improvement was defined as a composite endpoint of increased scores on both the MMSE and MoCA from baseline to follow-up. ALPS, cerebral hemisphere analysis along the perivascular spaces; Right ALPS index, right cerebral hemisphere ALPS; Left ALPS index, left cerebral hemisphere ALPS.

The right ALPS index demonstrated optimal performance in discriminating TBM from TBM-MCI patients (AUC = 0.719, 95% CI: 0.590–0.847, *p* = 0.003), with a sensitivity of 0.50, specificity of 1.00, and a Youden’s index of 0.50, as shown in Figure 6A.

A total of 29 TBM patients participated in the follow-up, with scanning intervals ranging from 6 to 12 months. All participants underwent two MMSE and MoCA assessments while drug-free and received standardized treatment according to clinical guidelines during the scanning period. Among them, 12 patients showed cognitive improvement, defined as an increase in scores on both the MMSE and MoCA from baseline to follow-up, while 17 patients were classified as not improved.

To identify predictors of cognitive prognosis, we first examined the univariate associations between all glymphatic imaging metrics (ALPS index, CPV, PVS) and the prognostic grouping (improved vs. not improved). The Left ALPS index was the only metric that showed a statistically significant difference between the two groups (*p* < 0.05). Based on this finding and its theoretical relevance to language and executive functions, we proceeded to evaluate its predictive performance using ROC analysis. The Left ALPS index demonstrated substantial prognostic value (AUC = 0.857, 95% CI: 0.638–1.000, *p* = 0.042), characterized by a sensitivity of 1.00, specificity of 0.714, and a Youden’s index of 0.714, as shown in Figure 6B. Other glymphatic metrics did not show significant predictive value and were not included in the ROC analysis to avoid multiple testing and overfitting.

#### 3.6.1 Distribution of change scores

Among the 29 patients who completed follow-up, the mean change in MMSE was +2.1 ± 3.4 points (range: −4 to +9), and the mean change in MoCA was +2.5 ± 4.1 points (range: −5 to +11).

#### 3.6.2 Sensitivity analysis for cognitive improvement

Using the more stringent definition (≥2-point increase on both MMSE and MoCA), all 12 patients classified as improved by the primary definition also met this criterion. Therefore, the sensitivity analysis yielded identical group classification (12 improved, 17 not improved), and the predictive performance of the left ALPS index remained identical to the primary analysis (AUC = 0.857, 95% CI: 0.638–1.000, p = 0.042; sensitivity = 1.00, specificity = 0.714). This perfect concordance demonstrates that our findings are highly robust and not dependent on the specific threshold used to define improvement.

#### 3.6.3 The exercise effect

Practice Effects: Although we did not have a healthy control group to formally assess practice effects, we note that the mean improvement observed in our patients (+2.1 points on MMSE, +2.5 points on MoCA) substantially exceeds the minimal practice effects reported in the literature for these scales (typically <1 point over 6–12 months in stable populations). Furthermore, the considerable heterogeneity in change scores (range: −4 to +9 for MMSE, −5 to +11 for MoCA) and the fact that baseline ALPS indices significantly predicted improvement argue against practice effects being the primary driver of the observed cognitive changes.

## 4 Discussion

TBM-MCI patients demonstrated a constellation of glymphatic and structural abnormalities, including a markedly reduced ALPS index, bilaterally increased diffusivity along projection and association fibers, enlarged choroid plexus volume, and region-specific vulnerability of the perivascular space network reflected by increased PVSVF-BG and decreased PVSVF-Hipp. These alterations were closely linked to clinical performance: lower ALPS values correlated with greater CPV enlargement and poorer cognitive outcomes across global cognition, executive function, and processing speed. Mediation analysis further identified right CPV as a full mediator between right ALPS and MMSE scores, indicating that CP inflammation constitutes a key pathway through which glymphatic impairment contributes to global cognitive decline. Together, these findings outline a coherent cascade from CP inflammation to glymphatic dysfunction and widespread white matter injury, ultimately leading to cognitive impairment in TBM-MCI. Moreover, this study provides the first demonstration of a reduced ALPS index in TBM, aligning with evidence from stroke and small-vessel disease and positioning glymphatic failure as a convergent mechanism across neuroinflammatory and neurovascular disorders.

Imaging markers suggest that perivascular clearance may be compromised in TBM, particularly with MCI. Mycobacterium tuberculosis-induced neuroinflammation may impair glymphatic flow via aquaporin-4 (AQP4) depolarization—a well-established mechanism of GD (15). Concurrently, characteristic TBM meningeal changes can physically obstruct CSF circulation, thereby indirectly impairing GF (42).

The CP, a key site for CSF production, BBB component, and neuroimmune hub (10), showed enlargement in the TBM, likely reflecting a robust local neuroimmune response to pathogen invasion (43). The negative correlation between the ALPS index and CPV strongly supports a causal role of CP dysfunction in driving GI (38, 43). We propose that inflammatory CPV enlargement impairs glymphatic clearance through altered CSF composition, BBB disruption, or disturbed pulsatile dynamics critical for waste clearance (44, 45). PVS is an established neuroimaging hallmark of impaired glymphatic and vascular clearance (46). In TBM-MCI, we observed a significant increase in PVSVF-BG alongside a reduction in PVSVF-Hipp. PVSVF-BG expansion typifies GD and serves as a recognized marker of impaired perivascular drainage and small vessel disease (2, 3). This aligns with evidence from other CNS disorders linking enlarged PVS to impaired waste clearance and vascular injury. Conversely, PVSVF-Hipp reduction presents a counterintuitive yet informative finding. We propose two mechanisms: local neuroinflammation may compress and occupy PVS-Hipp, or PVS collapse may reflect severe glymphatic inflow failure, both impairing solute clearance (47, 48). Given the hippocampus’s role in memory, these PVS alterations may underlie the memory deficits in TBM-MCI. Although CSF protein biomarkers (e.g., amyloid-*β*, tau, neurofilament light chain) were not measured in this study, previous work has demonstrated correlations between these biomarkers and DTI-ALPS indices in neurodegenerative disorders. The observed glymphatic imaging alterations in our TBM-MCI cohort may similarly reflect impaired protein clearance, a hypothesis that warrants direct testing in future CSF-integrated studies.

Altered diffusivity along y- and z-axes—corresponding to projection and association fibers—indicates microstructural damage from axonal injury, demyelination, or edema (49). The vulnerable hippocampus may undergo compression from severe inflammation/edema or reflect complex pathology like neuronal loss and gliosis (50). Together, these microstructural alterations likely represent downstream consequences of GI and neuroinflammation, linking vascular pathology to cognitive decline (51, 52). GD impairs clearance of metabolic wastes (e.g., amyloid-β, tau), promoting toxic accumulation that damages astrocytic end-feet, axons, and myelin (30, 38). These pathologies manifest as elevated diffusivity on DTI. Thus, selective tract abnormalities in TBM-MCI may reflect circuit-specific vulnerability, structurally underpinning characteristic cognitive deficits—notably slowed processing speed (53). CPV significantly correlated with multiple cognitive scores (MMSE, MoCA, SMDT, VFT, TMT), while reduced ALPS index associated with lower MMSE, consistent with prior reports (7, 54, 55). Recent evidence in PD and CSVD with MCI shows lower ALPS indices (8, 10), while longitudinal ALPS decline predicts MMSE performance in CSVD (9).

Mediation analysis revealed right CPV fully mediates the right ALPS index -MMSE relationship, positioning CP inflammation as an upstream driver of glymphatic failure (56). Mycobacterial infection likely triggers the release of pro-inflammatory cytokines (TNF-*α*, IL-1β, TGF-β) from CP, disrupting BBB integrity, altering CSF dynamics, and impairing AQP4-mediated clearance (13, 14). Partial mediation effects (Right CPV → Right ALPS index→TMTA; Right ALPS index→Right CPV → MMSE/TMTA) confirm GD as a key amplifier of CPV pathology. GD involves AQP4 depolarization (57, 58), perivascular protein accumulation (59), impaired arterial pulsation (60), and astrocytic metabolic deficit—collectively driving WM injury and executive/processing-speed deficits (61).

In fact, the interplay between Left ALPS index and right CPV may reflect hemisphere-specific vulnerability in TBM, implying that early lateralized glymphatic disruption could signal high-risk cognitive trajectories. Importantly, the absence of significant mediation by CPV on Hipp-PVS metrics implies that PVS alterations in the hippocampus may follow a parallel or independent pathway, perhaps reflecting neurotoxicity or neuronal loss rather than primary CP-driven glymphatic failure. Collectively, these results delineate a “CP-glymphatic-WM-cognition” axis in TBM-MCI, with CP serving as the trigger, the GS as the amplifier, and WM injury as the effector of cognitive decline. Recognition of this axis may open avenues for early imaging biomarkers and targeted therapeutic intervention. Right ALPS index best discriminates TBM from TBM-MCI, reflecting early asymmetric GI as a potential biomarker for early diagnosis. More importantly, the Left ALPS index predicts treatment prognosis, potentially guiding risk stratification and therapy adjustment. The innovation of our study lies in the fact that the ALPS index serves as a dual-purpose biomarker, being both sensitive to early impairment and predictive of progression. We first establish the glymphatic-cognitive relationship in TBM using a multi-parametric approach (ALPS, PVS, CPV), with automated PVS and CP quantification enhancing objectivity, reducing potential bias and enhancing clinical utility.

### 4.1 Interpretation of Glymphatic imaging markers

It is important to acknowledge that the imaging metrics used in this study—the DTI-ALPS index, PVS burden, and CP volume—are indirect proxies rather than direct measures of glymphatic function. While they are increasingly employed in the literature and have been validated against physiological measures in preclinical models, their translation to human studies requires cautious interpretation. The DTI-ALPS index, for instance, quantifies water diffusivity along the perivascular direction but does not directly measure the bulk flow of interstitial fluid or the clearance of metabolic wastes. It may be confounded by microstructural alterations in the white matter, such as axonal loss, demyelination, or edema, which are common in neuroinflammatory conditions like TBM. Similarly, PVS enlargement is a non-specific finding that can result from impaired perivascular drainage, but may also reflect vascular injury, inflammation, or even atrophy of the surrounding parenchyma. CP volume enlargement, while suggestive of neuroinflammation and barrier disruption, could also be influenced by age, vascular risk factors, or systemic inflammation.

Therefore, while our findings are consistent with the hypothesis of altered perivascular clearance and neuroinflammation contributing to cognitive impairment in TBM, they do not definitively establish glymphatic failure as the causal mechanism. Alternative explanations, such as direct vascular injury, inflammation-induced tissue damage, or axonal degeneration, may also contribute to the observed associations. Future studies combining dynamic contrast-enhanced MRI to directly visualize CSF tracer transport, or employing multi-compartment diffusion models to disentangle perivascular from parenchymal diffusion, would help validate and extend our findings.

In light of these considerations, we have used more cautious wording throughout the manuscript, referring to “imaging markers suggestive of altered perivascular clearance” rather than definitive “glymphatic dysfunction”.

Several limitations of this study should be acknowledged. First, while we used established screening tools and clinical criteria to classify MCI, the potential for misclassification remains a consideration in studies of this nature. The MMSE cutoff of 21, chosen to maximize sensitivity in this population, could theoretically introduce a risk of including participants with very early or prodromal dementia, despite our efforts to exclude dementia through clinical assessment of daily functioning. However, to empirically address this concern, we conducted a sensitivity analysis using a more conservative MMSE cutoff of 23, a threshold more commonly used to differentiate MCI from mild dementia. Importantly, this analysis revealed that all participants originally classified as TBM-MCI and TBM-nonMCI remained in their respective groups, with no reclassification occurring. This finding demonstrates the robustness of our grouping and suggests that any theoretical concern about misclassification at the lower MMSE boundary does not materially affect our cohort or conclusions. Nevertheless, future studies with longitudinal follow-up to confirm cognitive trajectories and exclude progression to dementia would further strengthen diagnostic certainty. Second, the cross-sectional nature of this study precludes causal inferences about the relationship between TBM and glymphatic dysfunction. Third, the small sample size warrants validation in larger cohorts. The cross-sectional design precludes causal inferences, necessitating longitudinal or animal studies. Fourth, ALPS, PVS, and CPV are indirect markers requiring validation against direct glymphatic measures like dynamic contrast MRI. Moreover, the ALPS index captures regional rather than whole-brain GF. Fifth, our CSF proteomics analysis was incomplete in protein coverage and lacked functional localization data. Finally, the mediation analyses presented in this study should be interpreted with caution due to the cross-sectional design. While mediation analysis can statistically disentangle the interrelationships among variables and test the consistency of hypothesized pathways, it cannot establish causal direction. The observed statistical mediation effects are consistent with our proposed “CP → ALPS → cognition” pathway, but alternative directional models (e.g., ALPS → CP → cognition) are also statistically plausible. Longitudinal studies or intervention trials are needed to determine the true temporal and causal sequence of these events. Future work using repeated measurements or experimental designs would help validate the directional interpretations suggested by our cross-sectional mediation findings.

## 5 Conclusion

In summary, this study establisheed a coherent model linking CP inflammation to GI, WM damage, and cognitive decline in TBM. These findings advance the understanding of the pathogenesis of TBM-MCI and highlight the ALPS index and CPV as promising diagnostic and prognostic biomarkers. Interventions aimed at the “choroid plexus-glymphatic” axis may represent a novel avenue for future research into preventing and treating TBM-related CI.

## Data Availability

The original contributions presented in the study are included in the article/Supplementary material, further inquiries can be directed to the corresponding author.

## Footnotes

1 FSL, http://www.fmrib.ox.ac.uk/fsl/

2
https://cabeen.io/qitwiki/

3 SPM12:https://www.fil.ion.ucl.ac.uk/spm/software/spm12/

4 v7.4.1, http://surfer.nmr.mgh.harvard.edu/

## Glossary

TBM: Tuberculous meningitis
CNS: Central nervous system
CI: Cognitive impairment
MCI: Mild cognitive impairment
GS: Glymphatic system
CSF: Cerebrospinal fluid
GF: Glymphatic function
DTI: Diffusion-tensor imaging
ALPS: Along the perivascular spaces
AD: Alzheimer’s disease
CSVD: Cerebral small vessel disease
PD: Parkinson’s disease
TBI: Traumatic brain injury
EPVS: Enlarged perivascular spaces
PVS: Perivascular spaces
CPV: Choroid plexus volume
BBB: Blood–brain barrier
GI: Glymphatic impairment
CP: Choroid plexus
HC: Healthy controls
MMSE: Mini-Mental State Examination
MoCA: Montreal Cognitive Assessment
TMT: Trail Making Test
CDT: Clock Drawing Test
VFT: Verbal Fluency Test
DST: Digit Span Test
RAVLT: Rey Auditory Verbal Learning Test
SDMT: Symbol Digit Modalities Test
TIV: Total intracranial volume
FA: Fractional anisotropy
WM: White matter
BG: Basal ganglia
Hipp: Hippocampal
PVSVF: PVS volume fraction
ROC: Receiver operating characteristic
AQP-4: Aquaporin-4
