# Supplementary material for: MRI markers of glymphatic dysfunction in tuberculous meningitis: associations with CSF proteins and cognitive impairment
Source: Front Neurol. 2026 Jun 5;17:1772389. doi: 10.3389/fneur.2026.1772389 (PMC13278861; doi:10.3389/fneur.2026.1772389)
Supplement: Supplementary file 1 [file Table_1.DOCX]

**SUPPLEMENTARY MATERIALS**

**CSF Proteomics (DIA) Analysis**

**Protein Extraction**
Frozen CSF samples were thawed on ice and suspended in protein lysis buffer (8 M urea, 1% SDS) supplemented with protease inhibitors. Samples were homogenized using a high-flux tissue grinder three times (180 s each), followed by non-contact cryogenic sonication for 30 min. After centrifugation at 16,000 g for 30 min at 8 °C, protein concentrations in the collected supernatants were determined using the bicinchoninic acid (BCA) assay (Thermo Scientific), following the manufacturer’s protocol. Protein quality was examined by SDS-PAGE.

**Protein Digestion**
A total of 100 µg of protein was resuspended in 100 mM triethylammonium bicarbonate (TEAB). Proteins were reduced with 10 mM tris(2-carboxyethyl) phosphine (TCEP) at 37 °C for 60 min and alkylated with 40 mM iodoacetamide (IAA) in the dark at room temperature for 40 min. After centrifugation at 10,000 g for 20 min at 4 °C, the pellet was resuspended in 100 µL of 100 mM TEAB. Trypsin was added at a 1:50 (enzyme:protein) ratio, and samples were incubated at 37 °C overnight.

**Peptide Desalting and Quantification**
Following digestion, peptides were dried using a vacuum concentrator, reconstituted in 0.1% trifluoroacetic acid (TFA), and desalted using HLB cartridges. Desalted peptides were dried again by vacuum concentrator and quantified using a NanoDrop One spectrophotometer (Thermo Scientific) based on UV absorbance.

**DIA Mass Spectrometry Acquisition**
Based on quantified peptide amounts, samples were analyzed using a Vanquish Neo UHPLC system coupled to an Orbitrap Astral mass spectrometer (Thermo, USA) at Majorbio Bio-Pharm Technology (Shanghai, China). Chromatographic separation was performed on a uPAC High-Throughput column (75 µm × 5.5 cm) with solvent A (water with 2% acetonitrile and 0.1% formic acid) and solvent B (80% acetonitrile with 0.1% formic acid). The LC gradient was set to 8 min. DIA data were acquired in Orbitrap DIA mode over an m/z range of 100–1700.

**Protein Identification and Quantification**
DIA raw files were processed using Spectronaut (version 19). Search parameters included a peptide length range of 7–52 amino acids, trypsin/P as the digestion enzyme, and a maximum of two missed cleavages. Carbamidomethylation of cysteine was set as a fixed modification, while methionine oxidation and N-terminal acetylation were defined as variable modifications. False discovery rate (FDR) thresholds for proteins and peptides were both set at ≤0.01, peptide confidence ≥99%, and XIC tolerance ≤75 ppm. Protein quantification was performed using the MaxLFQ algorithm.
Bioinformatic analysis was conducted on the Majorbio Cloud Platform. Differentially expressed proteins (DEPs) were identified using the R package “t-test,” with significance thresholds of fold change >1.2 or <0.83 and *p* < 0.05.

Supplementary Table 1: Sensitivity Analysis of Correlations Between Imaging Metrics and Cognitive Function After Additional Adjustment for Disease Severity and Structural Marker

| Imaging Metric | Cognitive Test | Original Partial Correlation (r) | Adjusted for MRC Stage+Lesion Score (r) |
| --- | --- | --- | --- |
| Left ALPS index | SDMT | 0.187* | 0.172* |
| Right ALPS index | MMSE | 0.186* | 0.168* |
| PVSVF-Hipp | MoCA | 0.295** | 0.271** |
| CPV (whole brain) | MMSE | -0.312** | -0.289** |

*p < 0.05, *p < 0.01; All models adjusted for age, sex, education, and TIV, with additional covariates as indicated.
